# Effect of *Aphis gossypii*-damaged *Alternanthera philoxeroides* on the biocontrol beetle *Agasicles hygrophila* (Coleoptera: Chrysomelidae)

**DOI:** 10.3389/fpls.2026.1867557

**Published:** 2026-07-29

**Authors:** Jisu Jin, Chen Lv, Fanghao Wan, Jianying Guo

**Affiliations:** 1 State Key Laboratory of Resource Insects, Institute of Apicultural Research, Chinese Academy of Agricultural Sciences, Beijing, China; 2 State Key Laboratory for Biology of Plant Diseases and Insect Pests, Institute of Plant Protection, Chinese Academy of Agricultural Sciences, Beijing, China; 3 Agricultural Genomics Institute at Shenzhen, Chinese Academy of Agricultural Sciences, Shenzhen, China

**Keywords:** *Agasicles hygrophila*, *Alternanthera philoxeroides*, *Aphis gossypii*, digestion and metabolism, volatile organic compounds (VOCs)

## Abstract

**Introduction:**

*Agasicles hygrophila* is a classical biocontrol agent widely used against the invasive weed *Alternanthera philoxeroides*. However, cotton aphid (CA) *Aphis gossypii* damage can alter host plant traits and reduce the fitness of *A. hygrophila*. The mechanisms underlying how aphid-damaged plant changes affect the behavior and physiological performance of this biocontrol agent remain unknown.

**Methods:**

We investigated the effects of CA-damaged alligatorweed on the development, nutritional metabolism, oxidative defense, digestive physiology, and host-selection behavior of *A. hygrophila*. Larval growth parameters, nutrient composition, oxidative defense-related and digestive enzyme activities, and digestion-related gene expression were analyzed after feeding on CA-damaged alligatorweed. Feeding and oviposition preferences were evaluated using choice experiments. Gas chromatography–electroantennographic detection (GC-EAD), gas chromatography–mass spectrometer (GC-MS), and electroantennogram (EAG) analyses were conducted to identify behaviorally active volatile organic compounds (VOCs), followed by behavioral responses that were analyzed use the Y-tube olfactometer assays.

**Results:**

Larvae feeding on damaged plants exhibited prolonged developmental durations and elevated mortality accompanied by diminished protein and lipid reserves but increased sugar content. Oxidative defenses were compromised, with significantly reduced activities of superoxide dismutase (SOD), catalase (CAT), and polyphenol oxidase (PPO). Digestive enzyme assays and qPCR analyses further revealed downregulation of trypsin and lipase activities, along with their encoding genes (*AhTRY* and *AhLPS*), whereas α-amylase activity and *Ahα-AMY* expression were markedly upregulated. Adult beetles showed a strong preference for feeding and ovipositing on undamaged plants over damaged ones. GC-EAD, GC-MS, and EAG analyses confirmed dose-dependent antennal responses to four VOCs. Behavioral assays demonstrated that oleonitrile, either alone or in combination with (+)-limonene, perillen or n-heptadecane, elicited strong repellency in adults.

**Discussion:**

These results indicate that CA-damaged alterations in plant chemistry impair larval metabolism and oxidative defense systems while releasing repellent VOCs that deter adults, thereby compromising *A. hygrophila’s* fitness and biocontrol efficacy against alligatorweed. These insights underscore the critical role of multitrophic interactions in refining biocontrol strategies for invasive weeds.

## 1 Introduction

Plants serve as fundamental components of terrestrial and aquatic ecosystems, acting as the primary food resource for heterotrophic organisms. Herbivorous insects represent a major threat to plant fitness, as they consume plant tissues, deplete nutrients, and transmit pathogenic microorganisms (Schopf and Hartl, 1997; Bak et al., 2013; Martins et al., 2019). To defend against herbivore attack, plants have evolved diverse protective strategies, among which induced plant defense is one of the most critical and well-studied mechanisms (Agrawal, 1998; Mithöfer and Boland, 2012; Hu et al., 2020; De Bobadilla et al., 2022). Induced defense refers to a series of physiological and biochemical modifications triggered by insect feeding, mechanical wounding, insect saliva, or herbivore-derived volatile organic compounds (VOCs) (Erb, 2009, 2012; Hare, 2011; Maffei et al., 2011; Dong et al., 2016; Erb and Reymond, 2019; Zhang et al., 2022a). Recent advances further reveal that plant defensive VOCs also interact with microbial communities in the phyllosphere and rhizosphere, forming a complex chemical communication network across plant, insect and microorganisms (Rizaludin et al., 2025). Once activated, complex intracellular signaling cascades are initiated, driving the synthesis and accumulation of diverse defensive compounds in host plants.

Plant defensive metabolites are generally divided into two categories: constitutive and induced defenses (Eigenbrode et al., 2008). Constitutive defenses are always present in plants, regardless of external stimuli, and they include physical barriers such as trichomes and lignin along with potentially altered secondary metabolites such as alkaloids and terpenoids (Jolivet and Bernasconi, 2006; Chen, 2008; Fuchs et al., 2017; Aljbory and Chen, 2018). In contrast, induced defenses are produced specifically in response to herbivore damage, mainly comprising VOCs such as methyl jasmonate (Holopainen and Blande, 2012), proteinase inhibitors (PIs) (Stout et al., 1999; Felton, 2005), lectins, and defense-related enzymes (Creelman and Mullet, 1997; Raj et al., 2006). Beyond protecting the host plant itself, herbivore-induced plant VOCs also act as interspecific signal molecules: they can activate defensive responses in adjacent undamaged plants or attract natural enemies of herbivorous insects, thereby forming a multi-trophic interaction network (Liu et al., 2015; Erb et al., 2015; de Lange et al., 2018; Hu et al., 2020; Timilsena et al., 2020; Jing et al., 2021; Lin et al., 2021; Gong et al., 2023; Yao et al., 2023).

The activation of plant-induced defense is regulated by sophisticated hormonal and genetic signaling pathways (Aljbory and Chen, 2018; Hu, 2019), with jasmonic acid (JA) (Halitschke et al., 2001; Schafer et al., 2011), salicylic acid (SA) (Maffei et al., 2007; Diezel et al., 2009; Benedetti et al., 2015; Khan et al., 2015; Ederli et al., 2020), and ethylene (ET) pathways (Kendall and Bjostad, 1990; O’Donnell et al., 1996; Schmelz et al., 2003) functioning as the core regulatory modules. Symbiotic microorganisms can also reshape these hormonal pathways and downstream defense traits, leading to differentiated plant resistance to herbivores (Martínez-Medina et al., 2024). Numerous studies have verified the effectiveness of such induced defenses in suppressing herbivore performance. For instance, the accumulation of proteinase inhibitors in tomato strongly inhibits the growth and survival of *Manduca sexta* (Halitschke et al., 2001), while VOCs released by damaged lima beans can recruit predatory mites and reduce pest populations (Ozawa et al., 2000).

In turn, herbivore-induced plant resistance also imposes profound impacts on the growth, development, and behavioral preferences of other insect species across trophic levels (Bröcher et al., 2025; Rivero et al., 2025). For example, transgenic *Bacillus thuringiensis* (Bt) and non-Bt cotton plants damaged by *Aphis gossypi* considerably influenced the oviposition preference of *Helicoverpa armigera* (Zheng et al., 2022). Similarly, *Tetranychus cinnabarinus*-induced cotton plants significantly influenced the development of *A. gossypii* (Ma et al., 2018). Moreover, multiple herbivores feeding on the same host often trigger antagonistic or synergistic plant defense responses, resulting in cascading effects on subsequent herbivore colonization and reproduction (Martínez-Medina et al., 2024; Rivero et al., 2025). Clarifying the reciprocal interactions between herbivorous insects and defense-induced plants is therefore essential for revealing the co-evolutionary relationships within plant-insect systems.

As one of the most notorious invasive weeds worldwide, alligatorweed (*Alternanthera philoxeroides* [Mart.] Griesb., Amaranthaceae) originates from South America and widely invades aquatic and semi-aquatic habitats including paddy fields, rivers and ponds (Buckingham, 1996). It is listed as one of the 16 most seriously invasive species in China and has been recognized as a prominent and problematic weed worldwide (State Environmental Protection Administration of China, and Chinese Academy of Sciences, 2003). *A. philoxeroides* aggressively competes with native flora for sunlight, nutrients, and water, often forming monocultures that displace indigenous plant communities in canals, riverbanks, and aquatic ecosystems. This process leads to irreversible alterations in community structure and ecosystem function (Holm et al., 1998; Ma and Wang, 2005; Shen et al., 2005). On terrestrial sites, it competes with agricultural crops, significantly reducing yields. In urban green spaces such as parks, residential areas, and roadside vegetation belts, it disrupts ornamental and ecological landscaping (Chang et al., 2013). In aquatic environments, dense mats of alligatorweed or mixed stands with other invasive macrophytes can reduce dissolved oxygen concentrations, causing hypoxia and subsequent mortality of fish, shrimp, and other aquatic organisms. These mats also serve as breeding habitats for mosquitoes and flies, posing additional public health risks (Quimby and Kay, 1977; National Invasive Species Council, 2001). Furthermore, the biomass accumulation can obstruct hydrological flow, clogging waterways and drainage systems (Julien et al., 1995).

Biological control is regarded as an eco-friendly and sustainable approach for invasive weed management. The flea beetle *Agasicles hygrophila* (Coleoptera: Chrysomelidae) is a classic specialist natural enemy of alligatorweed. Since its introduction to China in 1986, this beetle has achieved remarkable success in controlling alligatorweed spread across southern regions, representing one of the most successful weed biocontrol cases in China (Wang, 1989; Ma et al., 2003; Pan et al., 2006; Guo et al., 2012; Henriksen et al., 2018; Jin et al., 2021). Both adults and larvae of *A. hygrophila* feed exclusively on alligatorweed leaves, and mature larvae bore into stems for pupation; continuous feeding by field populations can rapidly defoliate and suppress alligatorweed growth.

One of the most common herbivores associated with alligatorweed is the cotton aphid, *Aphis gossypii* (CA, represents *A. gossypii*), a highly polyphagous phloem-feeding insect. It damages host plants by piercing and sucking phloem sap, leading to leaf deformation and declined crop productivity. Aphid feeding is well known to induce profound physiological and biochemical changes in host plants, including alterations in nutritional quality, defense-related pathways, and VOC emissions. Such changes may subsequently influence the performance and behavior of other herbivores feeding on the same plant. However, whether CA-damaged alligatorweed changes affect the biological control performance of *A. hygrophila* remains unknown.

During field surveys, we frequently observed *A. gossypii* infestations on alligatorweed and found that aphid feeding significantly alters the physicochemical properties of the host plant (*A. philoxeroides*) (**Supplementary Figure 1**), which severely compromises the effectiveness of *A. hygrophila* in controlling this invasive plant. Moreover, CA-damaged alligatorweed harbored fewer flea beetles and exhibited vigorous growth, whereas undamaged plants were severely consumed by *A. hygrophila* or even withered (**Supplementary Figure 2**). Studies have demonstrated that *A. philoxeroides* is susceptible to CA feeding (Henriksen et al., 2018), and the hooked epidermal hairs on plant leaves are lethal to *A. hygrophila* larvae, causing up to 100% mortality in the lower instars (Li et al., 2016). Indoor observation experiments revealed that *A. hygrophila* females preferred to feed on fresh, healthy leaves and lay eggs on leaves with normal morphology compared to that of the control group (**Supplementary Figure 3**). These observations suggest that CA-damaged alligatorweed may reduce the suitability of alligatorweed for *A. hygrophila*. However, the underlying mechanisms remain unclear. In particular, it is unknown whether CA-damaged changes in alligatorweed nutritional traits, defense-related responses, and VOC emissions influence the physiology, performance, host selection, and oviposition behavior of *A. hygrophila*. Addressing this knowledge gap is important for understanding multitrophic interactions in biological control systems and for improving the longterm management of alligatorweed.

Against this research background, we hypothesized that aphid infestation induces changes in the chemical and physiological traits of alligatorweed, thereby affecting both the behavioral preferences and physiological performance of the biological control agent *A. hygrophila*. Specifically, two hypotheses were tested. First, to test the hypothesis that VOCs emitted from CA-damaged alligatorweed influence host selection by *A. hygrophila* adults, resulting in altered feeding and oviposition preferences, behavioral assays and VOC analyses were conducted using CA-damaged and undamaged plants. Second, to test the hypothesis that CA-damaged changes in plant nutritional and defense-related traits reduce host suitability for *A. hygrophila* larvae, thereby affecting their growth, development, physiological biochemistry, and digestion-related gene expression, we analyzed the endogenous metabolites and physicochemical characteristics of CA-damaged alligatorweed and evaluated their effects on larval performance, enzyme activities, and digestion-related gene expression. The findings of this study will improve our understanding of plant-mediated interactions between two herbivorous insects sharing the same host plant and provide a theoretical basis for developing targeted and sustainable biological control strategies against invasive alligatorweed.

## 2 Materials and methods

### 2.1 Insects and host plants

#### 2.1.1 Insects

Adult leaf beetles of *A. hygrophila* were collected during the summer of 2020 in a pond field covered with alligatorweed near the Institute of Plant Protection, Hunan Academy of Agricultural Sciences (IPP, HAAS), Changsha, Hunan province, China (28°11′49′′N, 112°58′42′′E) using the sweeping method. These flea beetles were maintained on alligatorweed plants in the laboratory at the Institute of Plant Protection, Chinese Academy of Agricultural Sciences, Beijing, China (BJ, IPP, CAAS) and the Langfang Experimental Station of the Institute of Plant Protection, Chinese Academy of Agricultural Sciences (LF, IPP, CAAS) in Langfang City, Hebei Province under controlled conditions that were 26 ± 1 °C, 75 ± 5% relative humidity (RH), and a 12:12 h light–dark (L:D) regime (Guo et al., 2012). The flea beetles were cultivated for three generations to eliminate maternal effects prior to the experiments, and the sex of newly emerged adults (<12 h after eclosion) was identified based on the presence of a groove at the end of the abdomen.

*Aphis gossypii* were reared using the caging method described by Li and Han (2001). A gauze cage was constructed, and cotton aphids were fed separately on cultivated *A. philoxeroides* plants in the laboratory. The aphids were maintained in a climate-controlled chamber at 30 ± 1 °C, 75 ± 5% relative humidity, and a 12:12 light-dark photoperiod for multiple generations.

#### 2.1.2 Host plants

The roots of *A. philoxeroides* were collected from standing water at the IPP, HAAS, and planted in sterilized soil in plastic boxes (30 cm × 20 cm × 20 cm) in a greenhouse at the LF, IPP, CAAS, and BJ, IPP, CAAS, where they were watered daily. When the stems of *A. philoxeroides* reached 5-8 internodes, they were used for subsequent experiments. During the experiment, each *A. philoxeroides* plant was colonized with 100 A*. gossypii*.

### 2.2 Feeding and oviposition preference of *A. hygrophila* female adults

#### 2.2.1 Feeding preference assays

The feeding preference of female beetles for host plants was tested in Petri dishes (9 cm in diameter) in a constant-temperature incubator (RPX-450, Colin, Beijing) under controlled environmental conditions of 75 ± 5% relative humidity and a 12:12 light-dark photoperiod. One newly emerged adult female (<12 h after eclosion) was randomly selected and placed in a Petri dish lined with damp filter paper along with fresh *A. philoxeroides*. For each assay, a single fully expanded leaf of *A. philoxeroides* was collected from the same position of similarly sized plants and immediately used after excision. Prior to the experiment, leaves were briefly soaked in distilled water to maintain leaf turgor. The following treatments were applied: (a) undamaged alligatorweed leaves (control); (b) CA-damaged alligatorweed leaves; and (c) both undamaged and CA-damaged leaves presented simultaneously. For the dual-choice treatment, one undamaged leaf and one CA-damaged leaf were simultaneously presented on opposite sides of the Petri dish. The left-right positions of the two leaf types were randomly assigned for each replicate to minimize potential positional bias. All assays were conducted under identical environmental conditions. Leaves were replaced every 24 h, and their fresh weights before and after feeding were recorded using an analytical balance with a precision of 1/100000 (XS105DU, Mettler Toledo, Switzerland). The difference between initial and final leaf weight was used as an estimate of feeding consumption. Each treatment included 15 individual females, with each Petri dish representing one replicate.

#### 2.2.2 Oviposition preference assays

The oviposition preferences of *A. hygrophila* females were conducted in both Petri dishes (9 cm diameter) and mesh cages (60 × 40 × 40 cm) within a constant-temperature incubator (RPX-450, Colin, Beijing) under controlled environmental conditions. For the Petri dish assays, one newly emerged female (<12 h post-eclosion) and one male *A. hygrophila* were randomly selected and placed together in a Petri dish containing fresh alligatorweed. Leaf treatments followed the same protocol as described above. For the cage assays, five pairs of 1-day-old *A. hygrophila adults* were released into each mesh cage containing two alligatorweed plants: one undamaged plant (control) and one CA-damaged plant. The two plants were placed diagonally opposite each other within the cage at equal distances from the release point, allowing beetles to freely select their preferred host. To minimize potential positional bias, the locations of the undamaged and CA-damaged plants were randomly assigned among replicates. The number of eggs laid on each plant type (undamaged vs. CA-damaged) was recorded every 24 h until all females died. Each treatment was replicated three times.

### 2.3 Effect of feeding on CA-damaged alligatorweed on *A. hygrophila* adults longevity

Newly emerged (<12 h post-eclosion) male or female adults were randomly selected and individually placed into Petri dishes. Each dish was supplied with fresh *A. philoxeroides* corresponding to one of the two treatments described previously: (a) healthy *A. philoxeroides* and (b) *A. gossypii*-damaged *A. philoxeroides*. The *A. philoxeroides* were replaced daily, and adult survival was monitored daily until all individuals died. Each treatment consisted of three replicates, with 15 adults monitored per replicate.

### 2.4 Effect of feeding on CA-damaged alligatorweed on growth and development of *A. hygrophila* larvae

Fifteen newly hatched larvae (<12 h old) were randomly selected and individually placed in Petri dishes (diameter = 5.5 cm) containing fresh *A. philoxeroides*, prepared according to treatments a and b described in Section 2.2. The dishes were maintained in a constant-temperature incubator (RPX-450, Colin, Beijing) at 26 ± 1 °C, with 75 ± 5% relative humidity, and a 12:12 h light-dark photoperiod. Larval development was recorded daily until all individuals had either pupated or died. Each treatment was replicated three times, with 15 larvae per replicate.

### 2.5 Impact of CA-damaged alligatorweed on nutrient composition of *A. hygrophila* larvae

Larvae of *A. hygrophila* were fed detached undamaged alligatorweed leaves or CA-damaged alligatorweed leaves. The plant materials and feeding procedures were consistent with those described in Section 2.2, except that only single-choice feeding treatments were conducted. Larval samples were collected at 0, 24, 48, and 72 h after feeding. The protein, total sugar, and triglyceride contents were determined using a total protein (TPr) assay kit (Nanjing Jiancheng Bioengineering Institute, China, A045-4), a total sugar (TS) content assay kit (Solarbio Life Sciences, Beijing, China, BC2715), and a triglyceride (TG) content assay kit (Solarbio Life Sciences, Beijing, China, BC0625), respectively. Each treatment at each sampling time point included five biological replicates.

### 2.6 Effect of CA-damaged alligatorweed on antioxidant and digestive enzymes of *A. hygrophila* larvae

Samples were collected from flea beetle larvae at 0, 24, 48, and 72h post-feeding. The activities of two major antioxidant enzymes (superoxide dismutase, SOD; catalase, CAT) and polyphenol oxidase (PPO, an enzyme associated with oxidative defense and melanization) were measured using an SOD activity assay kit (Sangon Biotech Co., Ltd. China, D799594), a CAT activity assay kit (Sangon Biotech Co., Ltd. China, D799598), and a PPO activity assay kit (Sangon Biotech Co., Ltd. China, D799596), respectively. The activities of three major digestive enzymes, trypsin (TRY), lipase (LPS), and alpha-amylase (α-AMY), were measured using an TRY activity assay kit (Sangon Biotech Co., Ltd. China, D799678), a LPs activity assay kit (Sangon Biotech Co., Ltd. China, D799802), and a α-AMY activity assay kit (Sangon Biotech Co., Ltd. China, D799324). The experiments were conducted with five biological replicates.

### 2.7 Impact of CA-damaged alligatorweed on digestion-related gene expression in *A. hygrophila* larvae

To investigate the expression levels of digestion-related genes (*Trypsin*, *Lipase* and *alpha-amylase*) in *A. hygrophila* larvae following feeding on CA-damaged alligatorweed, larval samples were collected at 0, 24, 48, and 72h post-feeding on *A. philoxeroides* according to methods 2.2 a and b described above. Total RNA was isolated from the samples using the RNA extraction kit (UELandy Inc., China, UE-MN-MS-RNA-50). RNA concentration was measured using a NanoDrop spectrophotometer P330 (Implen, München, Germany). First-strand cDNA synthesis was performed using a reverse transcription kit (TransScript^®^ All-in-One First-Strand cDNA Synthesis SuperMix for qPCR (One-Step gDNA Removal). The cDNA templates obtained were used for RT-qPCR for gene expression. The target gene expression levels were assessed using a TransStart Green qPCR SuperMix Kit (Transgen, Beijing, China) on QuantStudio™ 1 Real-Time PCR Instrument (Applied Biosystems by Thermo Fisher Scientific), according to the manufacturer’s instructions. Specific primers of the three target genes were designed using Primer Premier 5. The primers for three genes were *AhTRY* (Forward primer: GTTCACTGTAGTCCTGCCG, Reverse primer: AAATCTGTTGGAATCTCTGG); *AhLPS* (Forward primer: GGTTTGCCAGCAGATACG, Reverse primer: AGGACAAAGACCTACTACCG); *Ahα-AMY* (Forward primer: GTTCCCTATCCTACCCAGC, Reverse primer: CCAGTTACACCCAAGTCG). The *β-actin* gene was used as an internal reference to normalize the gene expression, and relative expression levels were determined using the 2^–ΔΔCT^ method. Each experimental group included five biological replicates.

### 2.8 Plant volatile headspace collection

Dynamic headspace absorption was used to collect volatiles from *A. philoxeroides* plant samples (healthy vs. CA-damaged) as described by Chen et al. Healthy and CA-damaged alligatorweed plants were placed at the bottom of a 10 L glass jar during the growth period. A glass adsorption column (GAC) filled with 100 μg of Tenax TA adsorbent (80/100 mesh) was added to the jar, with gaskets at both ends to prevent the adsorbent from being lost. The experimental setup consisted of a glass adsorption column connected in series with an air pump (FCC-1500D, Yancheng Tianyue Instrument Co., Ltd, Jiangsu, China), a vial with activated carbon, a vial with distilled water, a glass sampling jar with plants, and a volatile trap containing the adsorbent. All components were connected to Teflon tubes and sealed with Parafilm to prevent air leakage. Prior to collection, charcoal-filtered fresh air was pumped through the glass jar for 60 min to remove air impurities from the system. The airflow was then adjusted to 1.2 L/min, and volatile collection commenced for 6 h. After collection, the GAC was immediately removed, and the volatiles were eluted twice from the adsorbent with 500 μL of n-hexane (HPLC Grade 4 L, Fisher, USA). Eluates were either stored at −20 °C or directly subjected to GC-EAD and gas chromatography-mass spectrometry (GC-MS) analyses. The volatile compounds in each plant treatment sample were replicated five times under identical conditions. Volatiles in an empty glass jar without plants were used as blank controls.

### 2.9 Volatile chemical analysis

Volatile components were analyzed and characterized utilizing a GC-MS system (GCMS-QP 2020, Shimadzu, Japan) equipped with an Agilent Rtx-5Sil MS (DB-5 MS) capillary column that possessed dimensions of 30 mm in length, 0.25 μm in film thickness, and 0.25 mm in inner diameter. The volume of sample injected was 1μL, and the injection port temperature was set to 250 °C in splitless mode. For the GC temperature increase procedure, the oven temperature was initially maintained at 50 °C for 2 min and then increased to 180 °C at 5 °C min^-1^, where it was held for 2 min. It was then increased to 250 °C at 10 °C min^-1^ and held for 3 min. Mass spectrometer (MS) analysis was performed in scan mode (m/z 35–450) with electron impact ionization (EI) at 70 eV. Volatile compounds were tentatively identified by comparing their mass spectra with the NIST 17.0 library database (NIST17-1, NIST17-2, and NIST17s) (Scientific Instrument Services, Inc., Ringoes, NJ, USA) and further confirmed by comparing their retention times with those of known authentic standards. Data acquisition and analysis were performed using GC-MS Solution 4.50 software (Shimadzu, Kyoto, Japan).

### 2.10 Candidates for olfactory active volatile organic compounds

To identify olfactory active volatile organic compounds (OAVOCs) from *A. philoxeroides* that elicit electrophysiological responses in the flea beetle *A. hygrophila*, we employed a gas chromatography system (GC, 7890A; Agilent Technologies, USA) coupled with electroantennographic detection (EAD, IDAC-2; Syntech, EU). Active volatile organic compounds (AVOCs) were further validated using electroantennogram (EAG) recordings. Prior to the experiment, a glass microelectrode was fabricated by inserting a silver wire into a capillary filled with a 0.9% NaCl solution. The head of a 1-day-old *A. hygrophila* adult was quickly excised from the base using a surgical blade under an anatomical microscope, and the tip of the antenna was removed (approximately 1 mm). Under a microscope, a reference electrode was inserted into the head of *A. hygrophila* and connected to an antennal potential system. Subsequently, the tip of the antennal club was connected to a micropipette on the recording electrode using a micromanipulator and fine forceps. The temperature parameters of the capillary column and column oven used for GC-EAD were the same as those used for GC-MS that facilitated OAVOC identification. A 1 μL sample of the volatile extract was split 1:1 and simultaneously analyzed by both flame ionization detection (FID) and EAD. Signals from the FID and EAD were processed using an IDAC-2 data acquisition controller, and data analysis was performed using GcEad 2014 software (Syntech, EU). To identify the AVOCs from *A. philoxeroides*, the GC-MS and GC-EAD spectra were integrated. Volatiles that triggered electrophysiological responses in the antennae were identified in the GC-EAD spectrum. The corresponding peaks were then located in the GC-MS spectrum based on retention time, position, and peak type. Finally, specific AVOCs were determined using the mass spectrum retrieval library NIST17.0.

To further assess the responsiveness of candidate compounds to the antennae, EAG (Syntech, The EU) tests were conducted to detect the dose-response relationship of biomarkers of individual AVOCs. A standard sample of each AVOC tested was diluted with n-hexane to prepare 10, 1, and 0.1 μg/μL solutions. 20 μL of each solution were then dripped onto a filter paper strip (5 cm long and 1 cm wide), and the filter paper was placed into a Pasteur pipette (long tip, 7 mm inner diameter, and 229 mm length) to serve as the odor stimulation source. n-Hexane was used as a control. The glass microelectrode and its connection to the antenna were prepared according to a previously described method. After the baseline EAG response became stable, a stimulus controller (CS-55, Syntech, The EU) was used to provide the odor stimulation air pulse through the Pasteur pipette and stimulate the antenna. EAG responses to 20 μL of n-hexane before and after the chemicals of single tested AVOCs were recorded. The sequence was as follows. First, the antenna was stimulated with n-hexane, and then, the chemicals of the single tested AVOCs were randomly selected and finally stimulated again with n-hexane. To ensure antennal sensitivity, at least a 30 s interval was maintained between every two stimulations. For all chemicals, there were 15 replicates each for male and female adults in the EAG tests.

### 2.11 Identification of key volatiles from CA-damaged alligatorweed that repel *A. hygrophila* adults

**Figure 1:**
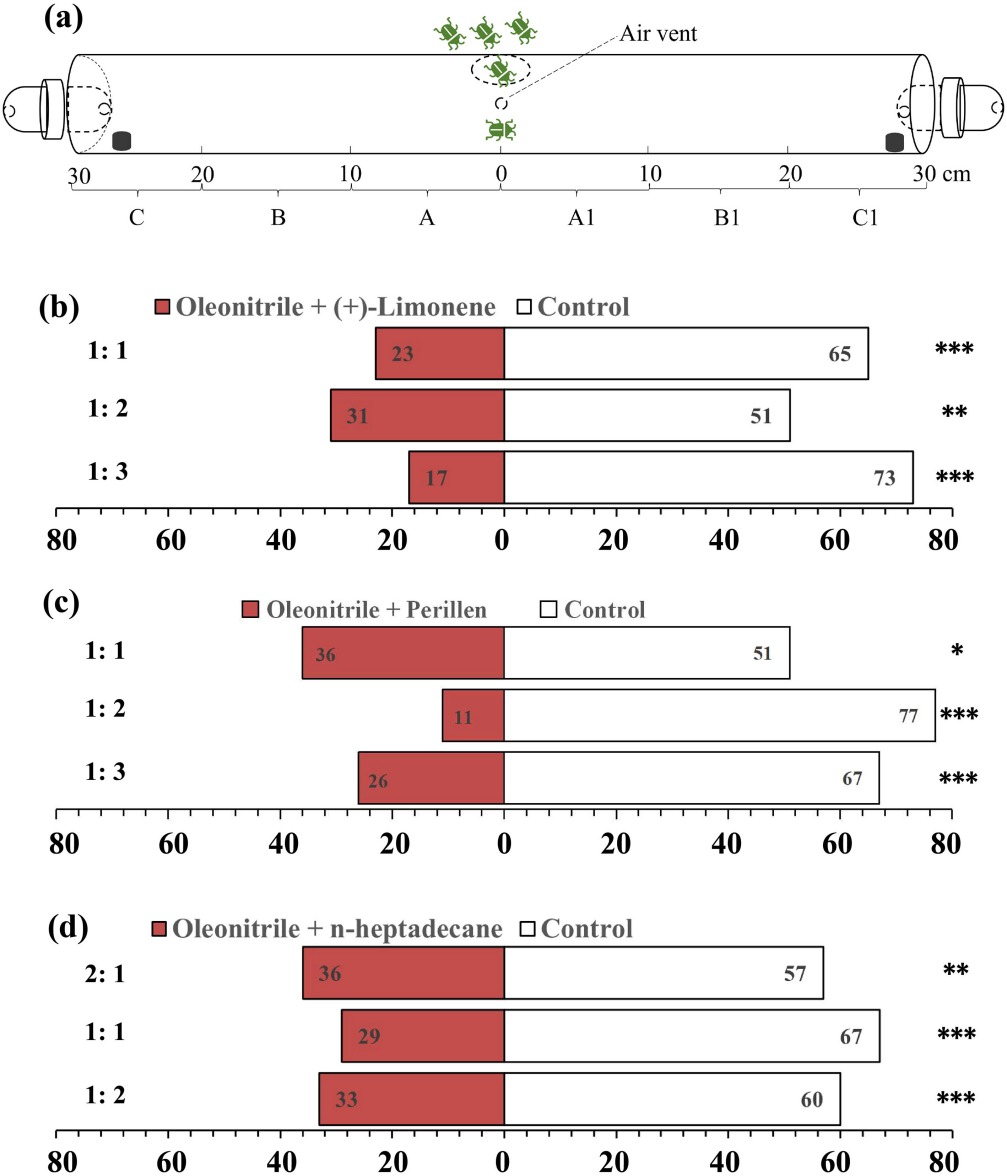
Behavioral responses of *Agasicles hygrophila* females to blended volatiles induced by CA-damaged at different ratios. **(A)** Apparatus used for the binary-choice behavioral assay; **(B)** Behavioral responses of *A. hygrophila* female to blends of oleonitrile and (+)-Limonene at different ratios; **(C)** Behavioral responses of *A. hygrophila* females to blends of oleonitrile and perillen at different ratios; **(D)** Behavioral responses of *A. hygrophila* females to blends of oleonitrile and n-heptadecane at different ratios. Behavioral responses in the Y-tube olfactometer assays were analyzed using the chi-square (χ²) goodness-of-fit test. “*” indicates a significant difference between treatments (*p < 0.05, **p < 0.01, ***p < 0.001).

Based on the EAG analyses, candidate alligatorweed volatiles (AVOCs) were selected for behavioral bioassays to evaluate their effects on *A hygrophila* adults using a binary-choice olfactometer (60 cm length, 3.5 cm internal diameter; **Figure 1A**). A centrally located circular hole (2.5 cm inner diameter) served as the insect introduction point. Compounds at different concentrations were formulated according to the manufacturer’s instructions. These standard samples were individually dissolved in n-hexane, and each compound was tested at three doses (10 μg/μL, 1 μg/μL, and 0.1 μg/μL). A 20 μL volume of the chemical was added dropwise to the filter paper strip with a pipette and connected to the behavioral device as the odor source, with n-hexane as the blank control. Prior to testing, beetles were starved for 4 hours, and all assays were conducted in complete darkness to eliminate visual cues. Additionally, to prevent odor contamination from previous assays, the glass olfactometer was rinsed with anhydrous ethanol and subsequently dried in an oven at 120 °C for 30 min. Because many plant volatile compounds are hydrophobic and may adsorb onto glass surfaces, ethanol was used to facilitate the removal of residual odorants. The olfactometer was then allowed to cool to room temperature before each assay to ensure complete evaporation of residual ethanol and to minimize any potential interference with insect behavioral responses. For each assay, five active adult beetles were sequentially introduced into the tube through a small circular hole, and the timing was initiated. Beetles were counted in the three test zones (A, B, and C; **Figure 1A**) after 10 min. Beetles remaining in zone A were considered non-responsive; those in zone B indicated an attractive or avoidance response; and those in zone C indicated a strong attractive or avoidance response. To minimize positional bias, the tube configuration was reversed after every two replicates. In addition to tests with individual compounds, behavioral assays were also performed using blends of AVOCs. A total of 100 male and female adults were used for behavioral testing.

### 2.12 Statistical analysis

Statistical analyses were performed using SPSS software v18.2 for Microsoft Windows. Data for *A. hygrophila* larval nutrient composition, enzyme activities, and digestion-related gene expression were analyzed using two-way analysis of variance (ANOVA) followed by Tukey’s honestly significant difference (HSD) test for *post hoc* multiple comparisons. For the no-choice feeding, oviposition preference assays and larval development parameters, differences between treatments were analyzed using Student’s *t*-tests. Data from dual-choice feeding and oviposition preference assays were analyzed using paired *t*-tests. Behavioral responses in the Y-tube olfactometer assays were analyzed using the chi-square (χ²) goodness-of-fit test to compare the observed frequencies of beetles choosing the treatment odor versus the control. Adult survival rates were analyzed using the log-rank (Mantel–Cox) test, and survival curves were generated using GraphPad Prism 8. Statistical significance was defined as *p* < 0.05 or lower.

## 3 Results

### 3.1 Feeding and oviposition preference of *A. hygrophila*

**Figure 2:**
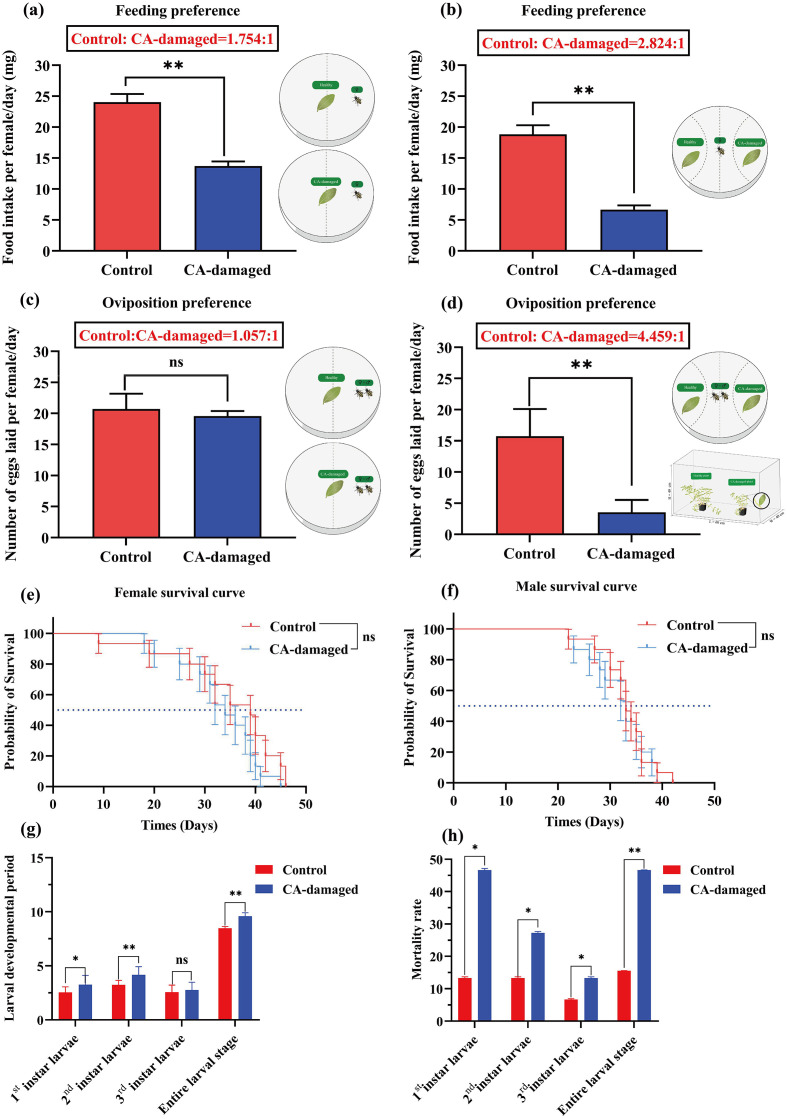
CA-damaged *Alternanthera philoxeroides* significantly affects feeding preference, oviposition preference, and larval development of the alligatorweed flea beetle *Agasicles hygrophila*. **(A, B)** Feeding preference of *A. hygrophila* females in no-choice and choice experiments, respectively. **(C, D)** Oviposition preference of *A. hygrophila* females in no-choice and choice experiments, respectively. **(E, F)** Effect of CA-damaged *A. philoxeroides* on the survival of *A. hygrophila* adults. **(G, H)** Effects of CA-damaged alligatorweed on the larval development and mortality rate of *A. hygrophila* larvae. Student’s *t*-tests were used for the no-choice feeding, oviposition preference assays and larval development parameters; paired *t*-tests were used for the dual-choice feeding and oviposition preference assays. All values are presented as the mean ± SD. “ns” indicates no difference between treatments, and “*” indicates a significant difference between treatments (*p < 0.05, **p < 0.01, ***p < 0.001).

In terms of feeding preference, *A. hygrophila* females exhibited a strong preference for undamaged alligatorweed plants under both choice and non-choice conditions. (non-choice test: *t* = 15.34, *p* < 0.001; choice test: *t* = 22.59, *p* < 0.001) (**Figures 2A, B**).

Regarding oviposition, there was no significant difference in the number of eggs laid on undamaged versus CA-damaged alligatorweed under no-choice conditions (*t* = 0.86, *p* = 0.423) (**Figure 2C**). However, when given a choice, females laid significantly more eggs on undamaged plants than on CA-damaged alligatorweed (*t* = 7.023, *p* = 0.006) (**Figure 2D**).

### 3.2 Effects of CA-damaged alligatorweed on larval development and adult longevity of *A. hygrophila*

The developmental period and mortality during the larval stage are presented in **Figures 2G, H**. Larval development was significantly affected by feeding on CA-damaged alligatorweed. Specifically, both the developmental duration and mortality rates of early instar larvae (first and second larval stages), as well as those across the entire larval stage, were significantly increased in individuals fed on CA-damaged alligatorweed. In contrast, no significant differences were observed in adult longevity for either sex (Female, χ^2^ = 2.383, *p* = 0.123; Male, χ^2^ = 0.274, *p* = 0.601) (**Figures 2E, F**).

### 3.3 Effect of CA-damaged alligatorweed on nutrient composition of *A. hygrophila* larvae

**Figure 3:**
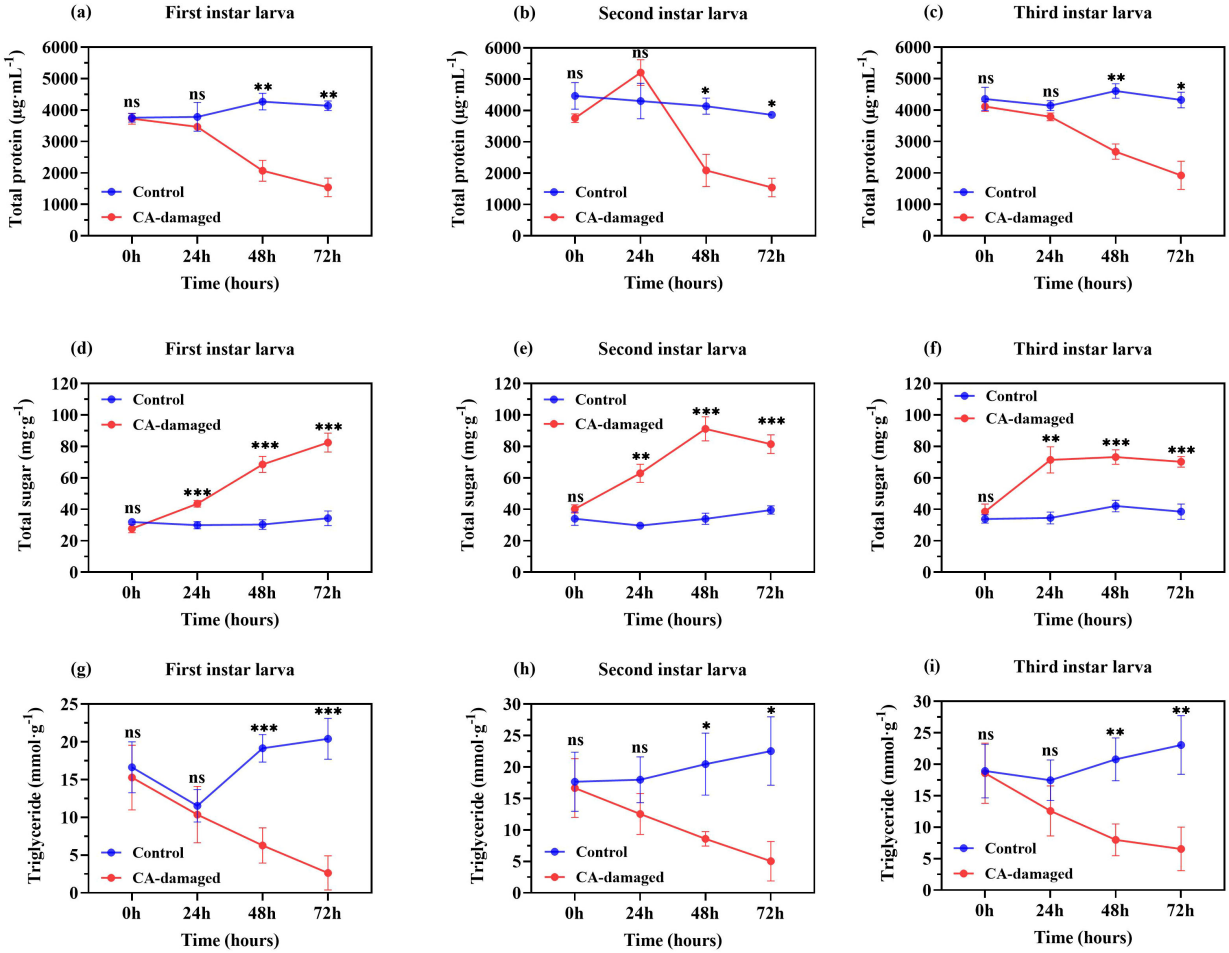
Impact of CA-damaged alligatorweed on nutrient composition in *Agasicles hygrophila* larvae. **(A–C)** Temporal changes in total protein content in first to third instar larvae after feeding on CA-damaged alligatorweed. **(D–F)** Temporal changes in total sugar content in first to third instar larvae after feeding on CA-damaged alligatorweed. **(G–I)** Temporal changes in triglyceride content in first to third instar larvae after feeding on CA-damaged alligatorweed. All values are presented as the mean ± SD. Data were analyzed using two-way ANOVA followed by Tukey’s honestly significant difference (HSD) test for *post hoc* multiple comparisons. “ns” indicates no difference between treatments, and “*” indicates a significant difference between treatments (*p < 0.05, **p < 0.01, ***p < 0.001).

Feeding on CA-damaged alligatorweed significantly affected the nutrient composition of *A. hygrophila* larvae compared to those fed on undamaged alligatorweed (**Figure 3**). For TPr, two-way ANOVA revealed significant effects of treatment (undamaged vs. CA-damaged alligatorweed), feeding time, and their interaction across all larval instars (First instar, F = 32.00, *p* < 0.001; Second instar, F = 29.37, *p* < 0.001; Third instar, F = 22.67, *p* < 0.001). Tukey’s HSD *post-hoc* test revealed showed that TPr contents did not differ significantly between treatments at 0 h and 24 h, whereas larvae fed on CA-damaged alligatorweed leaves exhibited significantly lower TPr levels at 48 h and 72 h compared with control (**Figures 3A, C**). Similarly, TG contents were significantly influenced by treatment, feeding time, and their interaction (First instar, F = 22.75, *p* < 0.001; Second instar, F = 14.83, *p* < 0.001; Third instar, F = 7.54, *p* = 0.002). No significant differences were detected at 0 h or 24 h; however, TG contents were significantly reduced in larvae fed on CA-damaged alligatorweed leaves at 48 h and 72 h across all larval instars (**Figures 3G–I**). In contrast, TS contents increased markedly following feeding on CA-damaged alligatorweed leaves. Two-way ANOVA detected significant effects of treatment, feeding time, and their interaction (First instar, F = 93.42, *p* < 0.001; Second instar, F = 55.59, *p* < 0.001; Third instar, F = 22.39, *p* < 0.001). *Post hoc* analyses indicated that TS contents became significantly higher in CA-damaged treatments from 24 h onward and remained elevated through 72 h in all larval instars (**Figures 3D, F**).

### 3.4 Effects of CA-damaged alligatorweed on oxidative defense-related and digestive enzyme activities in *A. hygrophila* larvae

**Figure 4:**
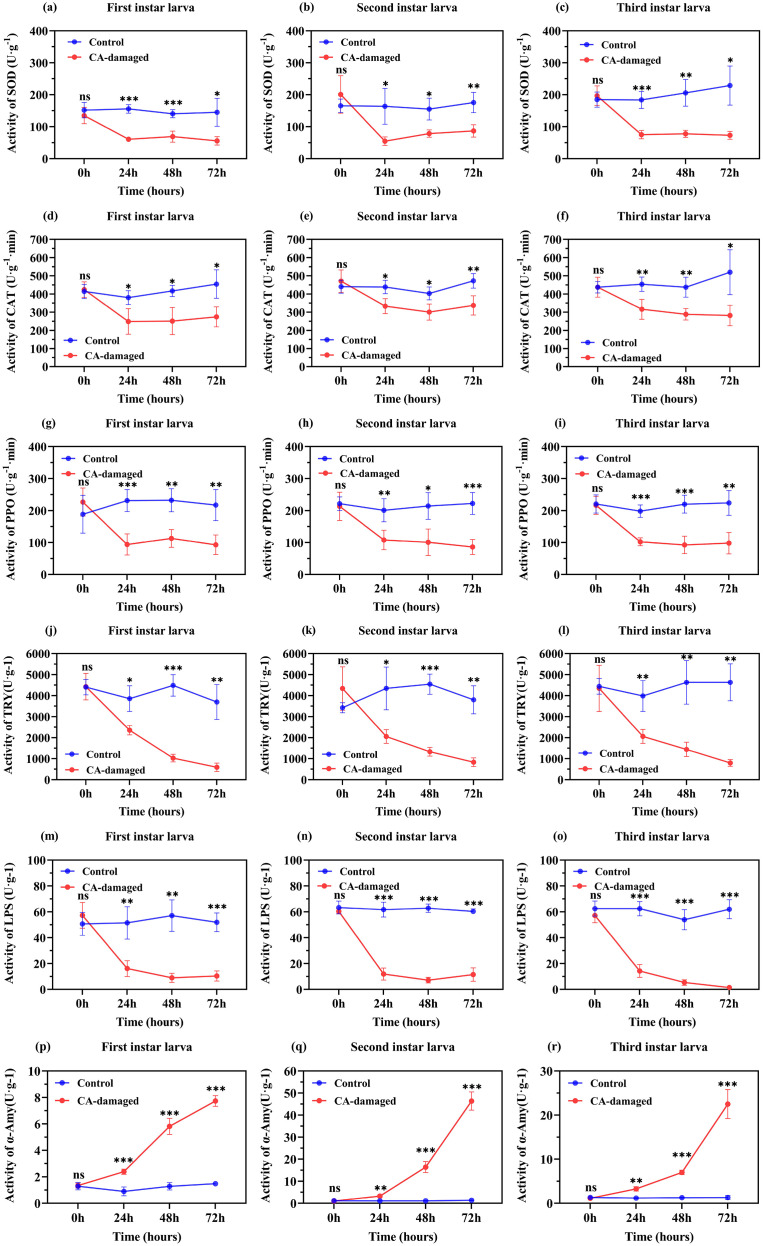
Effect of CA-damaged alligatorweed on oxidative defense-related and digestive enzyme activities in *A. hygrophila* larvae. **(A–C)** Superoxide dismutase (SOD) activity dynamics from the first to third instar after larvae fed on CA-damaged alligatorweed. **(D–F)** Catalase (CAT) activity dynamics across larval instars following feeding on CA-damaged alligatorweed. **(G–I)** Polyphenol oxidase (PPO) activity dynamics in larvae from first to third instar post-feeding on CA-damaged alligatorweed. **(J–L)** Dynamics of trypsin (TRY) activity from the first to third instar after larvae fed on CA-damaged alligatorweed. **(M–O)** Dynamics of lipase (LPS) activity across larval instars following feeding on CA-damaged alligatorweed. **(P–R)** Dynamics of alpha-amylase (α-AMY) activity in larvae from the first to third instar after feeding on CA-damaged alligatorweed. All values are presented as the mean ± SD. Data analyses were conducted using two-way analysis of variance (ANOVA), followed by Tukey’s HSD *post-hoc* test. “ns” indicates no difference between treatments, and “*” indicates a significant difference between treatments (*p < 0.05, **p < 0.01, ***p < 0.001).

Feeding on CA-damaged alligatorweed leaves significantly affected oxidative defense-related and digestive enzyme activities in *A. hygrophila* larvae, and these responses were strongly dependent on feeding duration (**Figure 4**). Two-way ANOVA revealed significant effects of treatment, feeding time, and their interaction for all measured enzymes (*p* < 0.05). Among the antioxidant-related enzymes, SOD, CAT, and PPO, an enzyme associated with oxidative defense and melanization, displayed equivalent activity levels across all experimental groups at the initial feeding time point but declined markedly in larvae fed on CA-damaged alligatorweed after 24 h. Such suppressive effects intensified gradually with extended feeding duration and were consistently detected across all larval instars (**Figures 4A–I**).

A similar pattern was observed for digestive enzymes involved in protein and lipid digestion. TRY and LPS activities were significantly inhibited in larvae fed on CA-damaged alligatorweed, with reductions becoming evident after 24 h and persisted for the entire subsequent feeding period (**Figures 4J–O**). In contrast, α-AMY activity exhibited the opposite trend, increasing gradually in response to feeding on CA-damaged alligatorweed and peaked at 72 h in all larval instars (**Figures 4P–R**).

### 3.5 Expression levels of digestion-related genes in *A. hygrophila* larvae following feeding on CA-damaged alligatorweed

**Figure 5:**
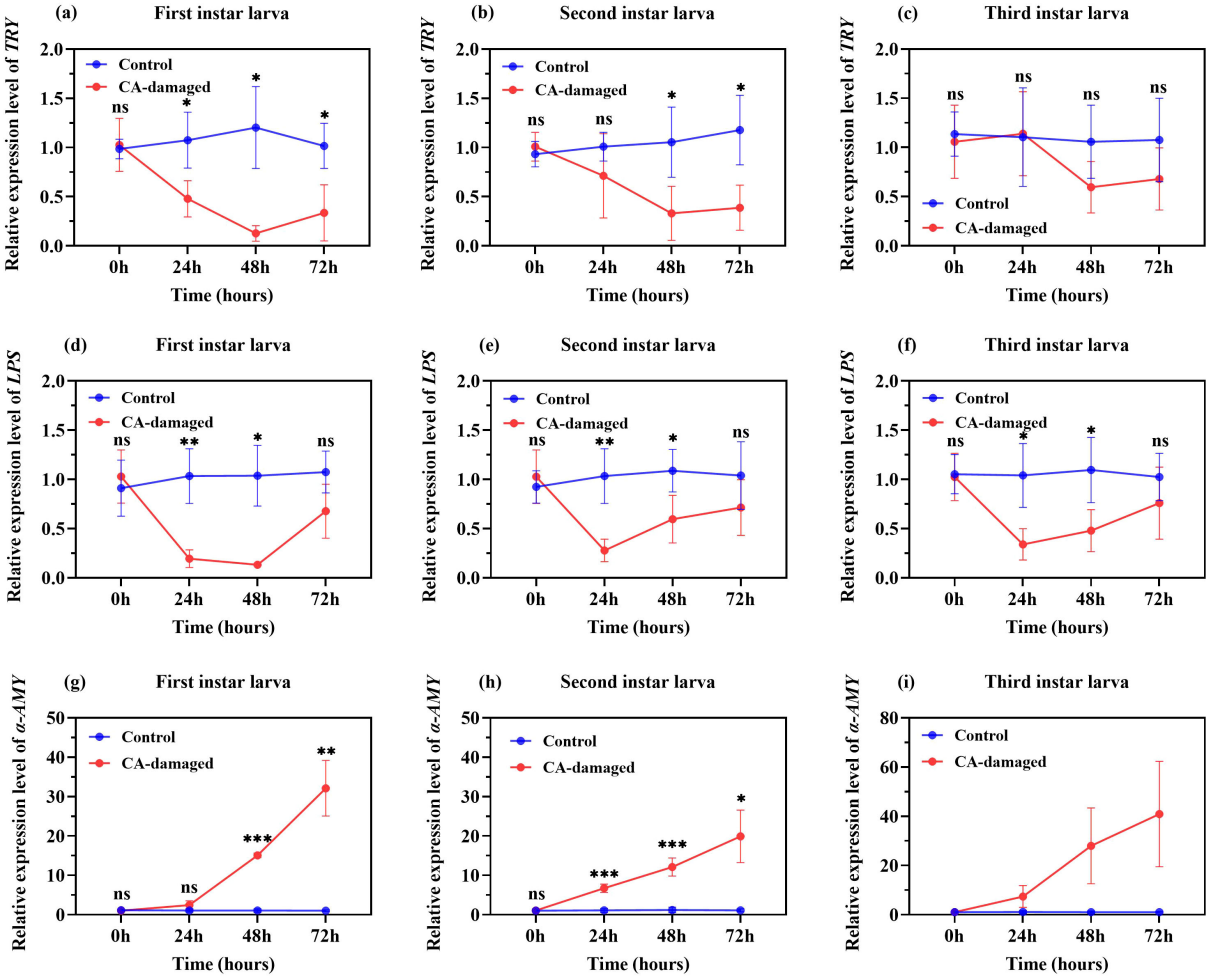
Effect of CA-damaged alligatorweed on the expression of digestion-related genes of *A. hygrophila* larvae. **(A–C)** Temporal expression patterns of trypsin gene (*AhTRY*) in first- to third-instar larvae after feeding on CA-damaged alligatorweed. **(D–F)** Temporal expression patterns of lipase gene (*AhLPS*) in first- to third-instar larvae after feeding on CA-damaged alligatorweed. **(G–I)** Temporal expression patterns of alpha-amylase gene (*Ahα-AMY*) in first- to third-instar larvae after feeding on CA-damaged alligatorweed. All values are presented as the mean ± SD. The figure shows data from five replicates that were analyzed. Data were analyzed using two-way ANOVA followed by Tukey’s honestly significant difference (HSD) test for *post hoc* multiple comparisons. “ns” indicates no difference between treatments, and “*” indicates a significant difference between treatments (*p < 0.05, **p < 0.01, ***p < 0.001).

The transcriptional levels of digestion-related genes in *A. hygrophila* larvae were significantly influenced by feeding on CA-damaged alligatorweed, and the magnitude of these responses varied among larval instars and feeding durations (**Figure 5**). Two-way ANOVA revealed significant effects of treatment, feeding time, and their interaction for genes examined (*p* < 0.05). The expression of *AhTRY*, encoding trypsin, exhibited a stage-dependent response to CA-damaged alligatorweed. For first- and second-instar larvae, *AhTRY* expression remained unchanged during the initial feeding period but decreased significantly at 48 h post-feeding compared with control (**Figures 5A, B**). In contrast, no significant change in *AhTRY* expression was observed in third-instar larvae (**Figure 5C**). A similar trend was observed for *AhLPS*, which encodes lipase. Compared with the control, *AhLPS* expression was significantly suppressed after 24 h of feeding on CA-damaged alligatorweed in all larval instars, although the inhibitory effect became weaker by 72 h (**Figures 5D–F**). Whereas the *Ahα-AMY* expression was significantly up-regulated in all three larval stages (**Figures 5G–I**). Collectively, these results demonstrate that feeding on CA-damaged alligatorweed reprograms the expression of digestive enzyme genes in *A. hygrophila*. This is consistent with the enzymatic responses observed in **Figure 4** and suggests a substantial shift in larval digestive metabolism following feeding on CA-damaged alligatorweed.

### 3.6 Identification of VOCs in CA-damaged alligatorweed

A comparative analysis of VOCs emitted by healthy and CA-damaged alligatorweed was conducted using GC-MS. A total of 19 VOCs were identified in healthy plants, and 23 VOCs were detected in the CA-damaged alligatorweed (**Supplementary Table 1**). These VOCs primarily consisted of terpenoids, aliphatic compounds, and aromatic compounds, with significant differences in the relative abundance of specific compounds observed among the different plant treatments. Furthermore, certain VOCs were observed exclusively in both healthy and CA-damaged alligatorweed, with 18 compounds common to both plants, one unique to healthy plants, and six unique to CA-damaged plants.

### 3.7 GC-EAD and EAG Screening for OAVOCs

Following GC-MS analysis, GC-EAD was used to identify five OAVOCs emitted by CA-damaged alligatorweed (**Supplementary Figure 4** and **Supplementary Table 2**). The identified OAVOCs comprised (+)-limonene, perillen, 1-dodecene, n-heptadecane, oleonitrile. Among these, n-heptadecane was detected in both healthy and CA-damaged alligatorweed, while the remaining four OAVOCs were exclusively found in CA-damaged alligatorweed.

**Figure 6:**
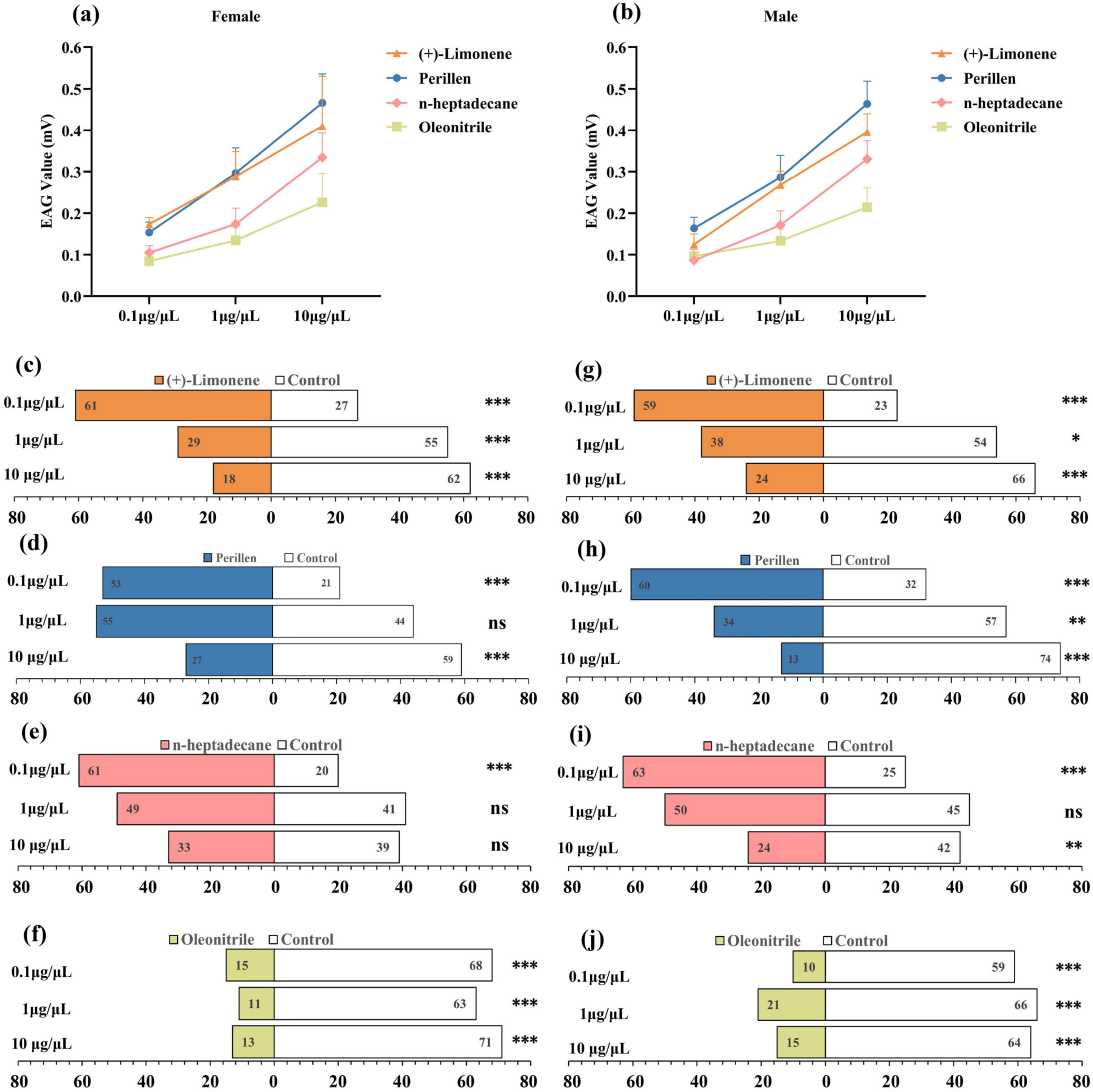
Antennal electrophysiological and behavioral responses of *Agasicles hygrophila* to *Alternanthera philoxeroides* volatiles induced by *Aphis gossypii* damage. **(A, B)** Dose-dependent electroantennography (EAG) responses of female and male *A. hygrophila* to five volatile compounds from *A. philoxeroides* induced by CA damage. **(C–F)** Behavioral responses of female *A. hygrophila* to four volatile compounds from CA-damaged alligatorweed. **(G–J)** Behavioral responses of male *A. hygrophila* to the same four volatile compounds. All values are presented as the mean ± SD. Electroantennogram recordings were analyzed using one-way ANOVA, and *post-hoc* comparisons were performed with Tukey’s HSD test. Behavioral responses in the Y-tube olfactometer assays were analyzed using the chi-square (χ²) goodness-of-fit test to compare the choice frequencies of beetles. Statistical significance is reported at *p* < 0.05 (*p < 0.05, **p < 0.01, ***p < 0.001).

EAG assays were conducted using standard compounds of the identified OAVOCs to evaluate antennal responses in *A. hygrophila* adults and to establish dose-response relationships (**Figures 6A, B**). Both male and female adults exhibited relatively strong EAG responses to (+)-limonene and perillen, while responses to dodecene was comparatively weaker. No significant differences in antennal responses were detected between sexes (**Supplementary Figure 5**). Dose-response relationships were established for the four OAVOCs that elicited EAG responses. The results demonstrated a dose-dependent increase in the EAG responses for most OAVOCs, with the exception of n-heptadecane (**Figures 6A, B**).

### 3.8 CA-damaged alligatorweed emitted VOCs that repel *A. hygrophila*

The behavioral responses of *A. hygrophila* adults to individual OAVOCs were investigated using a binary-choice olfactometer (**Figures 6C–J**). Overall, a high proportion of insects (82.6% on average) responded to the OAVOCs tested. Among the four OAVCs tested, (+)-limonene (Female, 10 μg/μL: χ^2^ = 48.400, *p* < 0.0001; 1 μg/μL: χ^2^ = 16.095, *p* < 0.0001; 0.1 μg/μL: χ^2^ = 26.273, *p* < 0.0001; Male, 10 μg/μL: χ^2^ = 39.200, *p* < 0.0001; 1 μg/μL: χ^2^ = 5.565, *p* = 0.018; 0.1 μg/μL: χ^2^ = 31.610, *p* < 0.0001) (**Figures 6C, G**), perillen (Female, 10 μg/μL: χ^2^ = 23.814, *p* < 0.0001; 1 μg/μL: χ^2^ = 2.444, *p* = 0.118; 0.1 μg/μL: χ^2^ = 27.676, *p* < 0.0001; Male, 10 μg/μL: χ^2^ = 85.540, *p* < 0.0001; 1 μg/μL: χ^2^ = 11.626, *p* = 0.001; 0.1 μg/μL: χ^2^ = 17.043, *p* < 0.0001) (**Figures 6D, H**), and n-heptadecane (Female, 10 μg/μL: χ^2^ = 1.000, *p* = 0.317; 1 μg/μL: χ^2^ = 1.422, *p* = 0.233; 0.1 μg/μL: χ^2^ = 41.506, *p* < 0.0001; Male, 10 μg/μL: χ^2^ = 9.818, *p* = 0.002; 1 μg/μL: χ^2^ = 0.526, *p* = 0.468; 0.1 μg/μL: χ^2^ = 32.818, *p* < 0.0001) (**Figures 6E, I**) exhibited dose-dependent effects on insect attraction. At high concentrations (10 μg/μL), these compounds repelled *A. hygrophila* adults, whereat lower concentrations (0.1 μg/μL), they attracted more insects than did the control. Conversely, oleonitrile consistently repelled *A. hygrophila* adults at all concentrations tested, indicating a potent deterrent effect for both male and female insects (Female, 10 μg/μL: χ^2^ = 80.095, *p* < 0.0001; 1 μg/μL: χ^2^ = 73.081, *p* < 0.0001; 0.1 μg/μL: χ^2^ = 67.687, *p* < 0.0001; Male, 10 μg/μL: χ^2^ = 60.785, *p* < 0.0001; 1 μg/μL: χ^2^ = 46.552, *p* < 0.0001; 0.1 μg/μL: χ^2^ = 69.594, *p* < 0.0001) (**Figures 6F, J**).

Based on the behavioral selection outcomes of the individual components mentioned earlier and the peak area ratios of the VOCs, we tested a two-by-two blend of oleonitrile and three other OAVOCs (perillen, [+]-limonene, and n-heptadecane) for their effects on *A. hygrophila* female behavior. More than 90% of the tested females responded to the blends. The results demonstrated that mixtures of oleonitrile with perillen, (+)-limonene, and n-heptadecane at different ratios consistently repelled *A. hygrophila* females compared to the control (oleonitrile + perillen, 1: 1: χ^2^ = 5.172, *p* = 0.023; 1: 2: χ^2^ = 96.023, *p* < 0.0001; 1: 3: χ^2^ = 36.151, *p* < 0.0001; oleonitrile + [+]-limonene, 1: 1: χ^2^ = 40.091, *p* < 0.0001; 1: 2: χ^2^ = 9.756, *p* = 0.002; 1: 3: χ^2^ = 69.689, *p* < 0.0001; oleonitrile + n-heptadecane, 2: 1: χ^2^ = 9.484, *p* = 0.002; 1: 1: χ^2^ = 30.083, *p* < 0.0001; 1: 2: χ^2^ = 15.667, *p* < 0.0001) (**Figures 1B–D**). These findings suggest that the presence of OAVOCs in combination with oleonitrile enhanced the repellent effect.

## 4 Discussion

Plants and herbivores exhibit complex and intricate relationships influenced by various biotic and abiotic factors that can either enhance or hinder their interactions (Li et al., 2023). Although this interaction is crucial in regulating ecosystem function, the specific mechanisms involved are not well understood. Through field surveys in Hunan and Henan provinces in China, we observed that CA-damaged alligatorweeds harbored fewer flea beetles compared to the healthy alligatorweeds in the same area. The damaged plants exhibited vigorous growth and were covered with needle-like hairs on the leaf surface (**Supplementary Figure 3**), whereas the healthy plants were heavily consumed by *A. hygrophila* and exhibited signs of withering. We propose that CA-damaged alligatorweeds develop defense mechanisms against herbivores by altering host traits such as nutrient content, antioxidant enzyme levels, and potentially altered secondary metabolites, ultimately reducing consumption by *A. hygrophila* (**Figures 2**, **3**). The fitness (reproduction and survival) of natural enemies is essential for biological control (Spencer, 1995). However, the effectiveness of biocontrol can be influenced by various factors, including biotic (soil microorganisms and other pests) (Caesar and Kremer, 2008) and abiotic factors (temperature, humidity, and light) (Zhao et al., 2016; Jin et al., 2021; Gerken et al., 2021). Although extensive research has focused on abiotic factors, exploration of biotic factors is relatively limited. In this study, we investigated the biotic factors of CA feeding-induced resistance in *A. philoxeroides* and their impact on its natural enemy, *A. hygrophila*.

Our experiments confirmed that *A. hygrophila* exhibited strong repellency towards CA-damaged alligatorweed. This discovery prompted subsequent experiments to investigate how CA-damaged alligatorweed affects the growth, development, and reproduction of *A. hygrophila* and how its VOCs affect the feeding and oviposition preferences of flea beetles. Research investigating aphid-damaged-induced plant immune responses has been a significant focus in entomological studies, particularly concerning physical, constitutive, chemical, and direct or indirect induced defenses in plants, aimed to understand plant defense mechanisms against aphid infestation (Wu and Baldwin, 2010).

Development and reproduction are crucial for the adaptation of herbivorous insects to host plants (Lu et al., 2016; Zhang et al., 2022b). However, to counter insect attacks, plants produce a myriad of specialized metabolites and proteins with repellent or antinutritional effects against the attackers (He et al., 2011; Kessler and Baldwin, 2001). The suitability and resistance of plants to herbivorous insects are closely linked to their nutritional content, defensive substances, and allelochemicals (Chen et al., 2009, 2023). Our study revealed that plants subjected to CA damage exhibited significantly lower levels of TPr and significantly higher levels of TS than that of healthy plants. Notably, these altered nutrient profiles were reflected in the larvae that fed on them. Furthermore, CA-feeding stimulated the activities of SOD, CAT, and PPO in *A. philoxeroides*, indicating enhanced antioxidant and defense-related responses in the plant. Conversely, *A. hygrophila* larvae exhibited significantly reduced antioxidant enzyme activity after consuming CA-damaged alligatorweed. Moreover, feeding on CA-damaged alligatorweed, the larvae of *A. hygrophila* exhibited significant alterations in both the enzymatic activities and gene expression levels associated with digestive metabolism. This suggests that feeding on *A. philoxeroides* damaged by *A. gossypii* disrupts the nutritional balance, digestive metabolism, and antioxidant capacity of *A. hygrophila* larvae, thereby compromising their fitness by ultimately impairing development and survival. It is crucial to note that high sugar levels can negatively affect insect growth, development, longevity, and antioxidant capacity. This is supported by studies demonstrating a 7% reduction in the median lifespan of *Drosophila melanogaster* fed sugar-rich diets (Dobson et al., 2017) and decreased lifespan, fertility, and antioxidant capacity in male *D. melanogaster* fed high-sucrose diets (Wen et al., 2022). These observations are consistent with our findings, further emphasizing the role of altered nutrient profiles in plant-herbivore interactions.

Additionally, in response to herbivorous insect feeding, plants release VOCs to directly repel or attract the natural enemies of herbivorous insects as a defense mechanism (Singh et al., 2021; de Bobadilla et al., 2022). For example, when cabbage plants (*Brassica oleracea*) are damaged by the herbivorous insect *Plutella xylostella*, they release VOCs that attract parasitoid wasp *Cotesia vestalis*, a natural enemy that parasitizes *Plutella xylostella*, thereby helping control its population (Girling et al., 2011). In this study, we observed that oleonitrile, a volatile compound of *A. philoxeroides* induced by CA feeding, exerted a significant repellent effect on *A. hygrophila* adults which, in turn influenced their feeding and oviposition preferences. Similar findings were observed in cotton plants damaged by *A. gossypii*, where the damaged plants exhibited a significant avoidance effect on the oviposition preference of *H. armigera* adults (Zheng et al., 2022). Although leaf water loss was not independently quantified, all treatments were subjected to identical handling and environmental conditions, thereby allowing reliable relative comparisons among treatments. As *A. philoxeroides* is a global invasive plant, this study will offer a reference for in-depth research examining the “feeding-defense” interactions between plants and herbivorous insects, elucidating the co-evolutionary relationship between plants and insects, and laying the foundation for the management of alligatorweed using *A. hygrophila* based on behavioral modulation techniques.

Insect host-search is not driven solely by olfactory cues; visual signals play a decisive role as well. Tansey et al. (2010) demonstrated that the cabbage seedpod weevil (*Ceutorhynchus obstrictus*) relies heavily on plant morphology and color for host recognition. When visual information was removed, adult weevils showed a marked decline in feeding activity, whereas restoration of visual stimuli promptly recovered feeding behavior. Their findings suggest that visual cues enhance host−location efficiency at several levels. In the present study we employed leaf discs as the odor source, which standardizes the chemical stimulus but inevitably removes the three-dimensional structure and color cues of the whole plant. Based on Tansey et al.’s observations, we hypothesize that incorporating intact host plants (thereby providing complete visual information) would enhance the foraging propensity of the *A. hygrophila* toward *A. philoxeroides*.

It is worth noting that previous studies have proposed that plant genotype, latitude, morphotype, and the invaded habitat are also key factors that shape *A. hygrophila* herbivory. The indirect effect of *A. gossypii* on *A. hygrophila* herbivory may differ among *A. philoxeroides* genotypes. For instance, genotypes with higher constitutive defenses (higher tannin or lignin content) might amplify the competitive pressure of aphids on flea beetles by further limiting nutrient availability, while genotypes with strong induced defense responses to aphid infestation could potentially reduce *A. hygrophila* feeding more significantly than the genotype used in our study (Williams et al., 2020; EPPO, 2024). Latitudinal climatic gradients may also modulate this tritrophic interaction. In high-latitude regions, lower temperatures and shorter growing seasons tend to reduce the developmental and feeding rates of both *A. gossypii* and *A. hygrophila*, potentially weakening their indirect competition. In contrast, low-latitude regions with warmer temperatures and longer growing seasons often support higher *A. gossypii* densities, which may intensify resource competition and lead to a more pronounced reduction in *A. hygrophila* herbivory than observed in our experiments (Sun et al., 2010; Dong et al., 2017). *A. philoxeroides* exhibits distinct morphotypes in aquatic versus terrestrial habitats aquatic morphs possess larger, thinner leaves with higher water content, while terrestrial morphs have thicker leaves with elevated fiber content (Fu et al., 2023) and these morphological differences may directly affect the feeding performance of *A. hygrophila.* In aquatic habitats, the high water content and low fiber content of *A. philoxeroides* leaves likely result in a relatively abundant allocation of soluble nutrients (sugars and amino acids), which may reduce the intensity of nutritional competition between aphids and flea beetles, thereby leading to a weaker indirect inhibitory effect of *A. gossypii* on *A. hygrophila* herbivory compared to our terrestrial experimental setting. In terrestrial habitats especially arid or nutrient-poor sites—*A. philoxeroides* allocates more resources to structural defenses (cellulose, lignin) at the expense of soluble nutrients. Under such conditions, aphid infestation can further deplete limited phloem nutrients, exacerbating resource limitation for *A. hygrophila* and strengthening the indirect negative impact on its herbivory, potentially exceeding the effect observed in our study (Fu et al., 2023). This speculation aligns with Henriksen et al. who demonstrated that hydrological conditions shape plant−insect interactions by modifying plant traits.

Unlike *A. hygrophila*, *A. gossypii* is a cosmopolitan polyphagous pest with minimal latitudinal distribution constraints. Distribution records show its presence across most of the world, from low-latitude tropical regions (Southeast Asia, the Amazon Basin) to high-latitude temperate and boreal zones (Northeast China, Northern Europe) (Ferrari et al., 2008), with no clear latitudinal boundaries. This cosmopolitan characteristic implies that *A. gossypii* may coexist with *A. hygrophila* and exert indirect interactions within the latitudinal range where *A. hygrophila* can survive; in high-latitude regions where *A. hygrophila* is absent, feeding by *A. gossypii* may directly affect *A. philoxeroides* growth, but interference with the biological control effect of *A. hygrophila* is not a concern.

It should be emphasized that our current study employed a single *A. philoxeroides* genotype. While controlled experiments provide mechanistic insights into the tritrophic interactions among *A. gossypii*, *A. hygrophila*, and *A. philoxeroides*, extrapolation to complex field conditions must be approached with caution. In natural ecosystems, additional biotic and abiotic factors may further modulate these indirect interactions. Despite these limitations, our findings highlight the importance of considering aphid-flea beetle indirect competition in the biological control of *A. philoxeroides*, especially in habitats experiencing high aphid pressure. This insight is broadly applicable to regions where both *A. gossypii* and *A. hygrophila* coexist on *A. philoxeroides*, and provides a reference for optimizing biocontrol strategies—for example, monitoring aphid populations to adjust the timing or density of *A. hygrophila* release.

## 5 Conclusion

**Figure 7:**
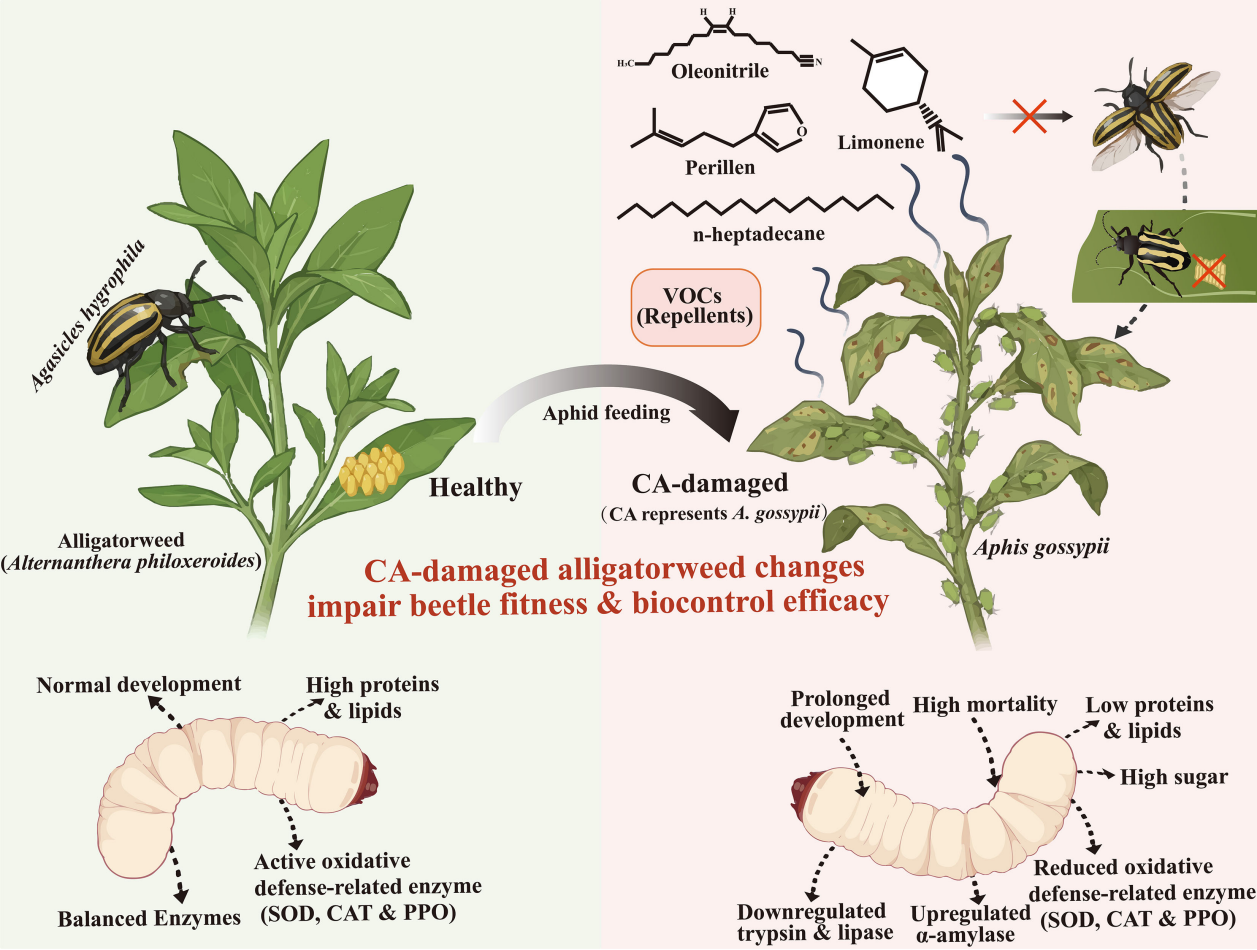
Summary of feeding by *Aphis gossypii* on *Alternanthera philoxeroides* reduces the fitness of *Agasicles hygrophila.* Plants damaged by *Aphis gossypii* become unsuitable for *A. hygrophila* adaptation due to altered nutrient composition, potentially altered secondary metabolites, and enhanced antioxidant enzyme activities. These changes disrupt the digestive metabolism and oxidative defense capacity of *A. hygrophila* larvae, resulting in prolonged larval development and increased larval mortality. Furthermore, oleonitrile—a volatile compound induced by *A. gossypii* feeding—exerted a deterrent effect on *A. hygrophila* adults, altering their feeding and oviposition preferences. Consequently, these factors lead to poor population levels of *A. hygrophila*.

In this study, we investigated whether feeding on *A. philoxeroides* by the *A. gossypii* affects the fitness of its natural enemy, the flea beetle *A. hygrophila*. The results demonstrated that *A. gossypii* feeding on *A. philoxeroides* significantly reduces the fitness of *A. hygrophila*, ultimately impairing its efficacy in controlling the invasive plant. Specifically, *A. gossypii* feeding markedly altered the physicochemical properties of *A. philoxeroides*, which in turn suppressed the digestive metabolism and oxidative defense capacity of *A. hygrophila* larvae. These physiological disruptions led to prolonged larval development and increased mortality. Additionally, oleonitrile—a volatile compound induced by *A. gossypii* feeding—exerted a deterrent effect on *A. hygrophila* adults, altering their feeding and oviposition preferences (**Figure 7**). Collectively, these findings reveal a significant reduction in the fitness of *A. hygrophila* and underscore the intricate interplay between invasive plants, herbivorous insects, and their natural enemies. This understanding is crucial for developing more effective biological control strategies for managing invasive alligatorweed populations.

## Data Availability

The original contributions presented in the study are included in the article/**Supplementary Material**. Further inquiries can be directed to the corresponding authors.
