# Supplementary material for: Effect of Aphis gossypii-damaged Alternanthera philoxeroides on the biocontrol beetle Agasicles hygrophila (Coleoptera: Chrysomelidae)
Source: Front Plant Sci. 2026 Jul 29;17:1867557. doi: 10.3389/fpls.2026.1867557 (PMC13480174; doi:10.3389/fpls.2026.1867557)
Supplement: Supplementary file 1 [file DataSheet1.docx]

**Supplementary Materials:**

**Supplementary Figure S1**: *Alternanthera philoxeroides* plants damaged by cotton aphid (CA) exhibited significant changes in their physicochemical properties. (a)-(c), Effects of *Aphis gossypii* feeding on the contents of nutrients (total protein, total sugar and total phenolics) in *Alternanthera philoxeroides*. (d)-(f), Effect of *A. gossypii* feeding on the activities of three defence enzymes (superoxide dismutase (SOD), catalase (CAT), and polyphenol oxidase (PPO)) in *A. philoxeroides*. (g), Effect of *A. gossypii* feeding on water content of *A. philoxeroides*. All values are shown as the mean ± SD. “ns” indicates no difference between treatments and “*” indicates a significant difference between treatments (* *p*< 0.05, ** *p*< 0.01, *** *p*< 0.001 by t-test).

**Supplementary Figure S2**: Feeding status of *Agasicles hygrophila* on *Alternanthera philoxeroides* with or without cotton aphid in the field. A and B: the number of *A. hygrophila* on *A. gossypii*-feed *A. philoxeroides* is low, the plant grows vigorously; C and D: non-*A. gossypii*-feed *A. philoxeroides* is severely fed by *A. hygrophila* or even died. (Photographed at Luohe, Xinyang, Henan Province, China on October 5, 2021)

**Supplementary Figure S3**: A, Phenotypic changes in leaves of healthy plants after feeding by *A. gossypii*; B, Identification of cotton aphid; C, Behavioral responses of volatiles from cotton aphids feeding on *A. philoxeroides* to the flea beetle *A. hygrophila*; D, Behavioral responses of the *A. hygrophila* to volatiles after excision of antennae.

**Supplementary Figure S4**: Antennal response of the flea beetles to *Alternanthera philoxeroides* volatiles. FID 1-3, Antennal response of the flea beetles to *Aphis gossypii*-damaged *Alternanthera philoxeroides* volatiles. FID 4, Antennal response of the flea beetles to healthy *Alternanthera philoxeroides* volatiles.

**Supplementary Figure S5**: Antennal responses of *Agasicles hygrophila* to *Alternanthera philoxeroides* volatiles. All values are shown as the mean ± SD. Different capital letters indicate the differences of antennal response of the same sex of *A. hygrophila* to different volatiles, and different lowercase letters indicate the differences of antennal response of the different gender of *A. hygrophila* to same volatile (*P*＜0.05, one-way ANOVA).

**Supplementary Table S1**: Identification of volatile organic compounds from healthy and *Aphis gossypii*-damaged *Alternanthera philoxeroides.* “-” means not detected. CK means clean plants, without herbivory and damaged.

| Number | Retention times | compounds | CAS | Relative Content (%) | |
| --- | --- | --- | --- | --- | --- |
|  |  |  |  | CK | CA- damaged |
| 1 | 8.824 | (+)-Limonene | 5989-27-5 | — | 10.34 ± 2.91 |
| 2 | 9.707 | Octanal | 124-13-0 | 0.32 ± 0.11 | 0.45 ± 0.06 |
| 3 | 10.686 | 1-Hexanol, 2-ethyl- | 104-76-7 | 0.18 ± 0.07 | — |
| 4 | 12.589 | Undecane | 1120-21-4 | 0.49 ± 0.13 | 0.61 ± 0.09 |
| 5 | 12.874 | Nonanal | 124-19-6 | 1.06 ± 0.03 | 2.13 ± 0.66 |
| 6 | 13.185 | (E)-4,8-Dimethylnona-1,3,7-triene | 19945-61-0 | 9.57 ± 0.41 | 7.77 ± 1.59 |
| 7 | 14.627 | perillen | 539-52-6 | — | 11.29 ± 3.13 |
| 8 | 15.921 | 1-dodecene | 112-41-4 | — | 1.35 ± 0.22 |
| 9 | 15.984 | Decanal | 112-31-2 | 0.53 ± 0.17 | 0.33 ± 0.11 |
| 10 | 16.05 | 2-Decen-1-ol, (E)- | 18409-18-2 | — | 0.37 ± 0.03 |
| 11 | 16.302 | Dodecyl aldehyde | 112-54-9 | 0.55 ± 0.25 | 0.72 ± 0.38 |
| 12 | 17.938 | n-heptadecane | 629-78-7 | 0.48 ± 0.31 | 5.57 ± 1.11 |
| 13 | 20.729 | Hexadecane, 2, 6, 10, 14-tetramethyl- | 638-36-8 | 0.93 ± 0.23 | 0.95 ± 0.20 |
| 14 | 21.953 | Eicosane | 112-95-8 | 0.80 ± 0.26 | 0.75 ± 0.15 |
| 15 | 22.528 | trans-alpha-Bergamotene | 13474-59-4 | 0.08 ± 0.01 | 0.10 ± 0.02 |
| 16 | 24.976 | 2,4-Di-tert-butylphenol | 96-76-4 | 0.83 ± 0.27 | 0.57 ± 0.04 |
| 17 | 25.091 | oleonitrile | 112-91-4 | — | 5.34 ± 1.46 |
| 18 | 26.342 | n-Tridecan-1-ol | 112-70-9 | 0.42 ± 0.07 | 0.56 ± 0.17 |
| 19 | 26.503 | cis-13-Docosenoamide | 112-84-5 | 0.27 ± 0.09 | 0.20 ± 0.03 |
| 20 | 26.710 | 2-Ethylhexyl 2-ethylhexanoate | 7425-14-1 | 1.00 ± 0.05 | 1.43 ± 0.17 |
| 21 | 28.757 | 9-Tricosene, (Z)- | 27519-02-4 | 0.24 ± 0.01 | 0.22 ± 0.02 |
| 22 | 29.54 | Benzoic acid, 2-ethylhexyl ester | 5444-75-7 | 0.08 ± 0.01 | 0.09 ± 0.02 |
| 23 | 31.241 | 1-Decanol, 2-hexyl- | 2425-77-6 | 0.58 ± 0.32 | 0.21 ± 0.06 |
| 24 | 34.48 | Carbonic acid, decyl heptadecyl ester | 112-17-4 | 0.70 ± 0.23 | 0.58 ± 0.32 |

**Supplementary Table S2**: Screening volatile organic compounds for *Agasicles hygrophila* antennae responses to *Alternanthera philoxeroides* based on GC-EAD

| Number | Retention times | English name | CAS |
| --- | --- | --- | --- |
| 1 | 8.824 | (+)-Limonene | 5989-27-5 |
| 2 | 14.627 | perillen | 539-52-6 |
| 3 | 15.921 | 1-dodecene | 112-41-4 |
| 4 | 17.938 | n-heptadecane | 629-78-7 |
| 5 | 25.091 | oleonitrile | 112-91-4 |
